# Weight-gain induced changes in renal perfusion assessed by contrast-enhanced ultrasound precede increases in urinary protein excretion suggestive of glomerular and tubular injury and normalize after weight-loss in dogs

**DOI:** 10.1371/journal.pone.0231662

**Published:** 2020-04-21

**Authors:** Daisy J. X. Liu, Emmelie Stock, Bart J. G. Broeckx, Sylvie Daminet, Evelyne Meyer, Joris R. Delanghe, Siska Croubels, Mathias Devreese, Patrick Nguyen, Evelien Bogaerts, Myriam Hesta, Katrien Vanderperren

**Affiliations:** 1 Department of Medical Imaging of Domestic Animals and Orthopedics of Small Animals, Faculty of Veterinary Medicine, Ghent University, Merelbeke, Belgium; 2 Department of Nutrition, Genetics and Ethology, Faculty of Veterinary Medicine, Ghent University, Merelbeke, Belgium; 3 Small Animal Department, Faculty of Veterinary Medicine, Ghent University, Merelbeke, Belgium; 4 Department of Pharmacology, Toxicology and Biochemistry, Faculty of Veterinary Medicine, Ghent University, Merelbeke, Belgium; 5 Department of Clinical Chemistry, Microbiology and Immunology, Faculty of Health Medicine and Life Sciences, Ghent University, Ghent, Belgium; 6 Oniris, National College of Veterinary Medicine, Food Science and Engineering, Nantes, France; University Medical Center Utrecht, NETHERLANDS

## Abstract

Early detection of obesity-related glomerulopathy in humans is challenging as it might not be detected by routine biomarkers of kidney function. This study’s aim was to use novel kidney biomarkers and contrast-enhanced ultrasound (CEUS) to evaluate the effect of obesity development and weight-loss on kidney function, perfusion, and injury in dogs. Sixteen healthy lean adult beagles were assigned randomly but age-matched to a control group (CG) (n = 8) fed to maintain a lean body weight (BW) for 83 weeks; or to a weight-change group (WCG) (n = 8) fed the same diet to induce obesity (week 0–47), to maintain stable obese weight (week 47–56) and to lose BW (week 56–83). At 8 time points, values of systolic blood pressure (sBP); serum creatinine (sCr); blood urea nitrogen (BUN); serum cystatin C (sCysC); urine protein-to-creatinine ratio (UPC); and urinary biomarkers of glomerular and tubular injury were measured. Glomerular filtration rate (GFR) and renal perfusion using CEUS were assayed (except for week 68). For CEUS, intensity- and time-related parameters representing blood volume and velocity were derived from imaging data, respectively. At 12–22% weight-gain, cortical time-to-peak, representing blood velocity, was shorter in the WCG vs. the CG. After 37% weight-gain, sCysC, UPC, glomerular and tubular biomarkers of injury, urinary immunoglobulin G and urinary neutrophil gelatinase-associated lipocalin, respectively, were higher in the WCG. sBP, sCr, BUN and GFR were not significantly different. After 23% weight-loss, all alterations were attenuated. Early weight-gain in dogs induced renal perfusion changes measured with CEUS, without hyperfiltration, preceding increased urinary protein excretion with potential glomerular and tubular injury. The combined use of routine biomarkers of kidney function, CEUS and site-specific urinary biomarkers might be valuable in assessing kidney health of individuals at risk for obesity-related glomerulopathy in a non-invasive manner.

## Introduction

Besides diabetes and hypertension, excess weight in the form of adipose tissue also increases the risk of developing obesity-related glomerulopathy and chronic kidney disease in humans [1]. Obesity-related glomerulopathy is characterized by glomerulomegaly, focal segmental glomerulosclerosis, hyperfiltration and subnephrotic to nephrotic-range proteinuria [2]. Weight-loss improves proteinuria and albuminuria in overweight and obese humans with chronic kidney disease [3]. Although the mechanism of how excess weight can lead to chronic kidney disease is not fully understood, physical (e.g., fat accumulation and ectopic fat in kidney), inflammatory (e.g., adipokines like leptin) and renal hemodynamic factors (e.g., increased glomerular filtration rate (GFR) and renal blood flow due to increased tubular sodium reabsorption) might play important roles [2].

Because of the kidney’s compensatory ability, obesity-related kidney injury can develop asymptomatically and might not be detected either by routine kidney function biomarkers (e.g., serum creatinine, blood urea nitrogen (BUN)) or by GFR measurements early in the disease [4]. Obesity-related glomerulopathy confirmation relies on renal biopsies, which are invasive [5]. Therefore, more sensitive and/or site-specific biomarkers of kidney injury and function that can aid in the early detection, monitoring, and potentially prevention of obesity-related glomerulopathy are needed [4]. Serum cystatin C (sCysC), for example, is a surrogate GFR marker less affected by external factors, e.g., muscle mass, than serum creatinine [6]. In dogs, biomarkers of glomerular injury, urinary immunoglobulin G (uIgG) and C-reactive protein (uCRP); and biomarkers of tubular injury urinary, retinol-binding protein (uRBP) and neutrophil-gelatinase-associated lipocalin (uNGAL), have shown potential to distinguish healthy animals from animals with kidney disease [7–10]. In humans, uIgG, uRBP, and uNGAL can detect diabetic nephropathy in an early stage [11].

Because microvascular disease might play a role in obesity-related glomerulopathy development, tools to evaluate early changes in renal microcirculation could also be helpful [12]. Contrast-enhanced ultrasonography (CEUS) utilizing gas-filled microbubbles enables non-invasive and -toxic real-time measurement of renal perfusion at both the macro- and microvascular level [13]. Furthermore, in humans, renal cortical perfusion assessed by CEUS parallels effective renal plasma flow [14]. In human studies, CEUS could distinguish those in early stages of chronic kidney disease from healthy volunteers [13]. In a murine model of obesity-related glomerulopathy, CEUS detects increased cortical perfusion time, accompanied by a decrease in cortical microvessel density [15].

The aim of this study was to use CEUS and a set of selected sensitive and site-specific renal biomarkers to assess when and how changes in kidney function, perfusion and injury occur during gradual diet-induced obesity and subsequent weight-loss in dogs. Plasma clearance of exo-iohexol was used as a gold standard for renal function. Although animal models demonstrate that obesity can cause kidney injury and dysfunction [15–18] and human studies show that weight-loss can improve kidney function [3,19,20], to our knowledge, no study has examined kidney function and injury during the development of obesity and after weight-loss in the same individual yet. Furthermore, using dogs, which have been previously used as models for the study of diet-induced obesity and the kidney [17,21–23], allows for a highly controlled study that is otherwise difficult to perform with human subjects.

## Methods

### Animals and experimental protocol

This longitudinal study of approximately 1.5 years was approved by the Institutional Animal Ethics Committee (Faculties of Veterinary Medicine and Bioscience Engineering, Ghent University, Belgium; EC2016/92) and performed in accordance with European (European Directive (2010/63/EU)) and national guidelines for the care and use of animals. Sixteen lean purpose-bred adult beagles (mean (standard deviation (SD)), 4.4 (2.0) years; six intact and two spayed females, four intact and four neutered males) were housed in controlled kennel conditions (12:12 h light-dark cycle). All dogs were considered healthy based on their medical history, on physical examination, complete blood count, serum biochemistry profile, abdominal ultrasonography, routine urinalysis (sediment examination, dipstick analysis, specific gravity measurement), urine protein-to-creatinine ratio (UPC) and urine bacterial culture.

After adapting to a commercial adult maintenance dry diet (Veterinary^™^ HPM Adult Large and Medium, Virbac, Carros, France) for four weeks, the dogs were equally divided into two groups, randomly, matched by age and sex. In the control group, eight beagles were fed to maintain a lean body weight and body condition score (BCS 4-5/9) for 83 weeks [24]. Part of the dataset from these eight beagles were published by Liu et al [25,26]. The body condition scoring system based on 9-points used in this study is a validated method to semi-quantitively assess the body composition of dogs base on visual and palpable characteristics [24]. The system ranges from 1 which stands for cachectic to 9 which stands for severely obese. A BCS of 4 or 5 represents an ideal body composition [24]. Individual maintenance energy requirements, based on the National Research Council requirement of 552 kJ/kg^0.75^, were adjusted when needed to maintain lean body weight [27]. Based on the protocol of a previous canine obesity study [28], the other eight beagles of the weight-change group were initially fed 1.3 x maintenance energy requirements using the same diet as the control group to gradually induce obesity (week 0 to 47) and adjusted when needed. Overweight was defined as having a BCS of 6 or 7/9 and obesity as a BCS of 8 or 9/9 (ref. 24). From week 47 to 56, the amount of food was initially reduced 10%, and hereafter adjusted to maintain a stable body weight. From week 56 to 83, the weight-change group was initially fed resting energy requirements (293 kJ/kg^0.75^, based on week 0 body weight as ideal body weight) to induce weight-loss. The amount was adjusted weekly to maintain a gradual weight-loss rate of 0.5–2% per week. The analyzed nutrient composition of the diet is shown in S1 Table. The dogs were fed individually once a day and had free access to water. Food intake was recorded daily. body weight and BCS were evaluated weekly.

**Fig 1:**
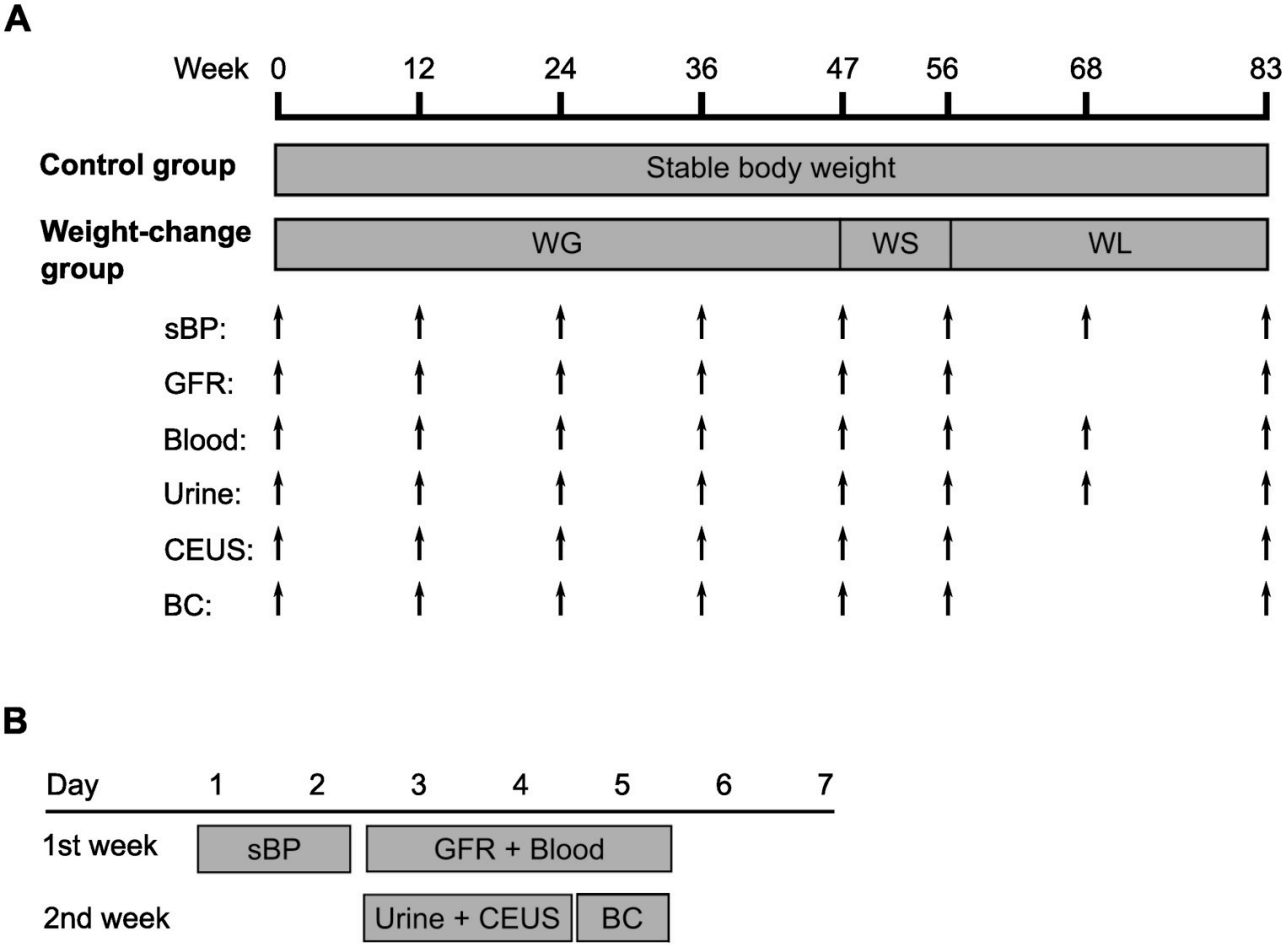
Schematic representation of the study protocol. (A) Summary of the sample points and timeline of the procedures used during the entire study for both the control and the weight-change groups. The control group was fed to maintain a stable body weight. The weight-gain group was fed to gain weight (WG), to remain at a stable weight (WS), and to lose weight (WL) in three consecutive phases. The procedures performed were measurement of systolic blood pressure (sBP), glomerular filtration rate (GFR), blood and urine collection, contrast-enhanced ultrasonography (CEUS) and body composition analysis (BC). (B) Timeline of the procedures during each sample point, except for week 68 when all procedures were performed within 3 days, starting measurement of sBP and ending with blood and urine collections.

Measurements were made after the adaptation period at week 0, 12, 24, 36, 47, 56, 68 and 83 and performed over two weeks. A schematic representation of the study protocol is shown in Fig 1. During the first week of each time point, systolic blood pressure (sBP) was measured on the first over two days in the first week. The next three days dogs were fasted and blood samples (12 mL) were collected from the jugular vein (21G needle), and GFR was measured. On the third and fourth day of the second week, morning urine samples (10 mL) were collected by ultrasound-guided cystocentesis (22G needle) without sedation, followed by CEUS under sedation. On the last day of the week body composition was determined. All are methods described below. During week 68, only sBP, blood sampling and cystocentesis were performed and completed within 3 days. Complete blood count and serum biochemistry (Architect C16000, Abbott Max-Planck-Ring, Wiesbaden, Germany) were repeated at week 24, 47, 56 and 83. Serum creatinine concentrations were considered normal when <1.4 mg/dL [29]. BUN values were considered normal when between laboratory ranges (6–57 mg/dL). Blood samples were centrifuged at 2000 × *g* for 5 min at 21°C within 2 h of collection and serum divided into 250 μL aliquots for sCysC and serum leptin. An aliquot of morning urine (5 mL) was used for urinalysis (dipstick analysis, specific gravity, UPC (Iricell IQ; Instrumentation Laboratory, Zaventem, Belgium); sediment analysis (IQ 200 SPRINT, Instrumentation Laboratory, Zaventem, Belgium); and bacterial culture). A UPC value greater than 0.5 was defined as proteinuria [29]. Another 5 mL of urine was centrifuged at 450 × *g* for 3 min at 21°C. The supernatant was stored in 200 μL aliquots within 30 minutes of collection for urinary biomarkers. Both serum and urine samples were stored at -80°C. The dogs were not sacrificed at the end of the study.

#### Measurement of systolic blood pressure and GFR

sBP was measured indirectly using the Doppler technique (Parks Medical Electronics Inc., Aloha, OR, USA) on the right forelimb according to the recommended procedures of the consensus statement of the American College of Veterinary Internal Medicine [30]. Systemic hypertension was defined as sBP > 150 mmHg [30].

After a 12 h fast, GFR was measured (mGFR) by determination plasma clearance of exo-iohexol after an intravenous bolus administration of iohexol (64.7 mg/kg body weight, Omnipaque 300^®^, GE healthcare, Diegem, Belgium), as previously described [10,31]. Plasma was divided into 300 μL aliquots and stored at -20°C. Clearance of exo-iohexol was determined by noncompartmental analysis using Phoenix 6.4 (Princeton, NJ, USA).

#### CEUS examination and quantitative analysis

Prior to CEUS examinations, dogs were fasted for 12 h. CEUS under sedation (0.4 mg/kg, Dolorex^®^ 10 mg/ml, MSD, Mechelen, Belgium) was performed on all dogs in a standardized manner, following the protocol described in Liu et al [25]. Briefly, the same linear transducer (12–5 MHz) and settings (single focus directly under the kidney, image depth 5 cm, persistency off, mechanical index 0.08, dynamic range C50, gain 85%, frame rate 8 Hz, and side-by-side imaging) on a dedicated machine (iU22) with contrast-specific software were used for all contrast-enhanced ultrasound evaluations. The transducer was held manually in a longitudinal plane on the kidney and in the same position. All ultrasound imaging was performed by one of the co-authors (E.S.). Commercially available sulfur hexafluoride-filled microbubbles (SonoVue^®^, Bracco Diagnostics Inc., Milan, Italy) were prepared and administered as a bolus (0.04 mL/kg) following the manufacturer’s guidelines (D.L. and K.V.). Microbubbles were injected into a catheter through a three-way stopcock, followed immediately by 2 mL of sterile saline. Simultaneously with the injection, the timer was set at 0 and a 90-s digital recording was made. Between each injection, microbubbles were destroyed by scanning the caudal abdominal aorta at a mechanical index of 0.5. The left kidney was imaged first and twice, followed by the right kidney, which was imaged once. A bolus was repeated if a large movement artifact was present. The recording from the second bolus of the left kidney was used for further evaluation [32]. An example of a recording can be seen in S1 Video.

**Fig 2:**
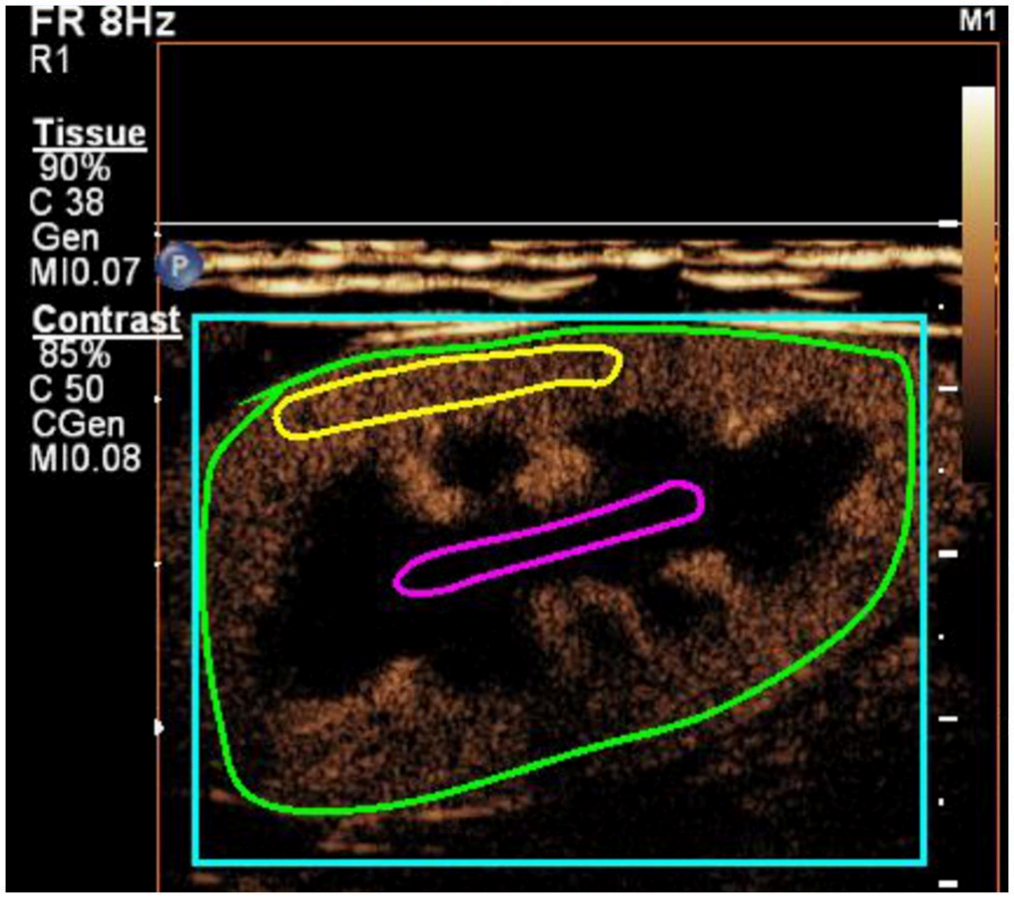
A contrast-enhanced ultrasound image of a left kidney in the sagittal plane of dog with region-of-interests drawn in the renal cortex (yellow line), renal medulla (magenta line) and on the entire kidney (green line). The size of region-of-interests, from which time-intensity curves were generated, were 0.51 cm^2^ for the cortex and 0.37 cm^2^ for the medulla.

**Fig 3:**
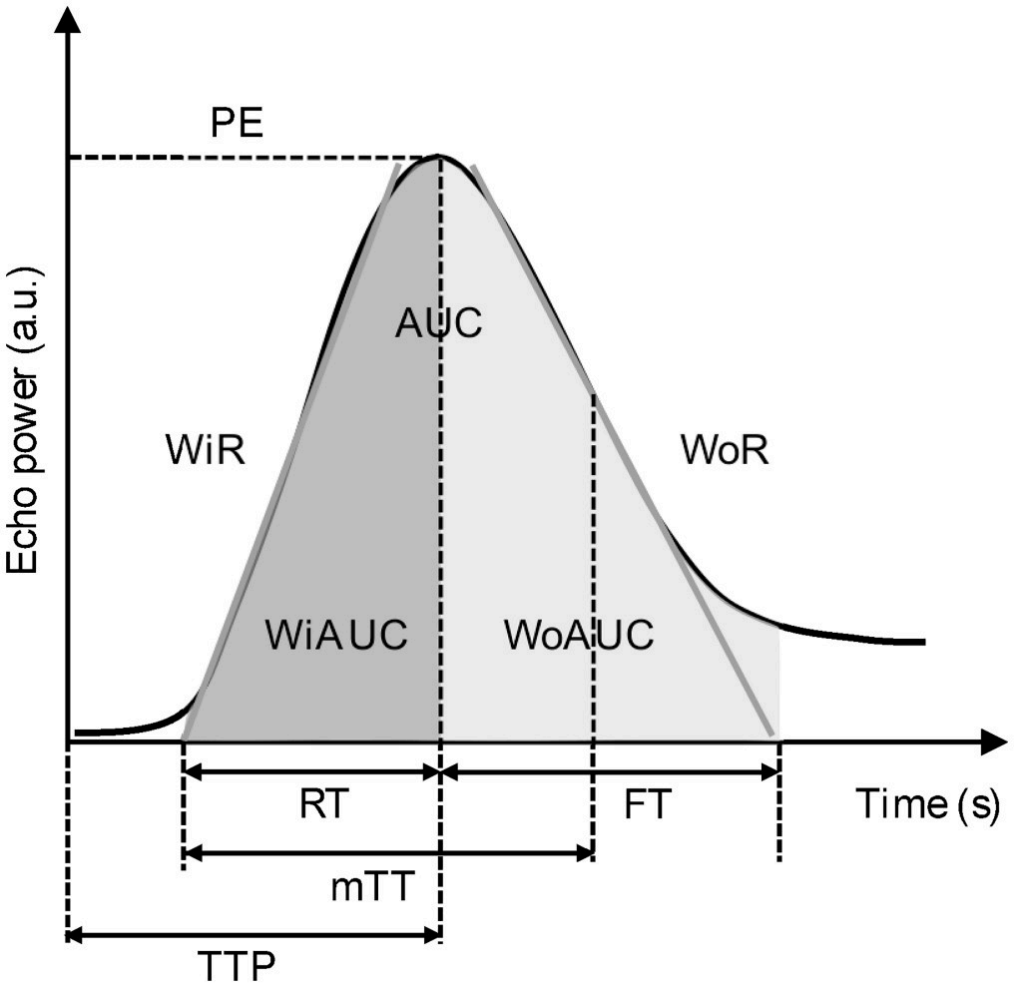
Schematic representation of a time intensity curve of the kidney. The x-axis represents the time in seconds (s) and the y-axis represents the intensity in arbitrary units (a.u.). Intensity-related parameters are as follows: peak enhancement (PE) referring to maximum enhancement; wash-in area under the curve (WiAUC) and wash-out area under the curve (WoAUC) referring to the sum of all amplitudes inside the range from the beginning of the curve up to the time-to-peak (TTP) and from the TTP to the end of the descending curve, respectively; and total area under the curve (AUC)). Time-related parameters are as follows: rise time (RT) referring to the time interval between the first arrival of contrast and TTP; mean transit time (mTT) representing the mean duration of complete contrast perfusion, refers to the time when the tissue begins to enhance with contrast medium until the enhancement is half of the PE; TTP referring to the time of contrast agent injection to maximum enhancement; fall time (FT) referring to the duration of contrast wash-out) were determined from the time intensity curves. The slopes of the time-intensity curve are wash-in (WiR) and wash-out rate (WoR) referring to the maximum and minimum slopes, respectively. Wash-in perfusion index (WiPI) is calculated as WiAUC divided by RT. Adapted from the VueBox user manual with permission [33].

The CEUS clips were analyzed using specialized computer software (VueBox, Bracco Suisse, Switzerland) for objective quantitative analysis by first author. The quantification protocol was previously described by Liu et al [25]. Briefly, three region-of-interests (ROI) were manually drawn, keeping the area and depth for each location approximately the same for every dog at all time points (Fig 2). The software generated time-intensity curves and determined mean pixel intensities for each ROI. Intensity-related parameters representing blood volume and time-related parameters representing blood velocity were determined from the time-intensity curve. The definition for each parameter is presented in Fig 3.

#### Body composition determination

Body composition was determined by isotopic dilution of deuterium oxide using Fourier-transform infrared spectroscopy as previously described [28,34]. Samples were centrifuged at 2000 × *g* for 5 minutes at 21°C and plasma stored in 300 μL aliquots at -20°C.

#### Determination of serum leptin, sCysC and urinary biomarkers

Following manufacturer’s instructions, serum leptin concentration was measured using a validated, commercially available canine ELISA kit (Millipore Corp., Billerica, MA, USA) in one batch [35]. The limit of detection and of quantification for this kit was 1.04 ng/mL and 1.88 ng/mL, respectively.

Immunoassays for sCysC, uCRP, uIgG, uNGAL and uRBP have been described in detail elsewhere [26]. The concentration of each urinary biomarker was expressed as a ratio to urinary creatinine (/c) to account for variations in urine concentration [36].

### Statistical analysis

For the immunoassays, statistical handling of data from samples below the limit of detection or between the limit of detection and of quantification were published in Liu et al [26].

A stepwise analysis was conducted. First, associations between body characteristics (body fat % and lean mass; dependent variables) and independent variables serum leptin, markers of kidney function and kidney injury, and CEUS renal perfusion parameters were analyzed within the weight-change group using a linear mixed model with dog as random effect. Variables with significant associations (P < 0.05) with either of the body characteristics were used next in a linear mixed model where the significance of the interaction between group and time was evaluated to compare both groups over time. For the CEUS parameters, kidney depth was accounted for in the mixed models (fixed effect). Dog and kidney side were included as random effects. When the interaction was significant, a post hoc comparison for each time point was conducted and a Bonferroni correction was consistently applied to account for multiple testing. The results are presented as mean ± SD for body characteristics, sBP, serum leptin, serum creatinine, BUN, mGFR, UPC, sCysC and the urinary biomarkers; and for the CEUS parameters of the cortex and medulla of both groups or median (minimum–maximum) for BCS. All statistical analyses were conducted in R version 3.4.4. In all mixed models, the default variance-covariance structure of the lme4-package was used.

## Results

One of the control dogs was diagnosed with multicentric lymphoma 1 month after the week 56 measurements and was euthanized. Necropsy confirmed the diagnosis of multicentric lymphoma with pulmonary and hepatic involvement, but there were no signs of lymphoma infiltration in the kidney. Therefore, data from this dog were kept for statistical analysis. At week 24, urine collection and CEUS was not possible for another control dog because it was briefly hospitalized for injury unrelated to the study (i.e., eye trauma).

### Body characteristics

At week 0, the control group and weight-change group dogs weighed 11.6 ± 1.7 kg and 11.2 ± 2 kg, and had 14.6 ± 6.1% and 15.8 ± 7.1% body fat, respectively. They had a lean BCS of 4 (4–4 and 4–5, respectively).

**Fig 4:**
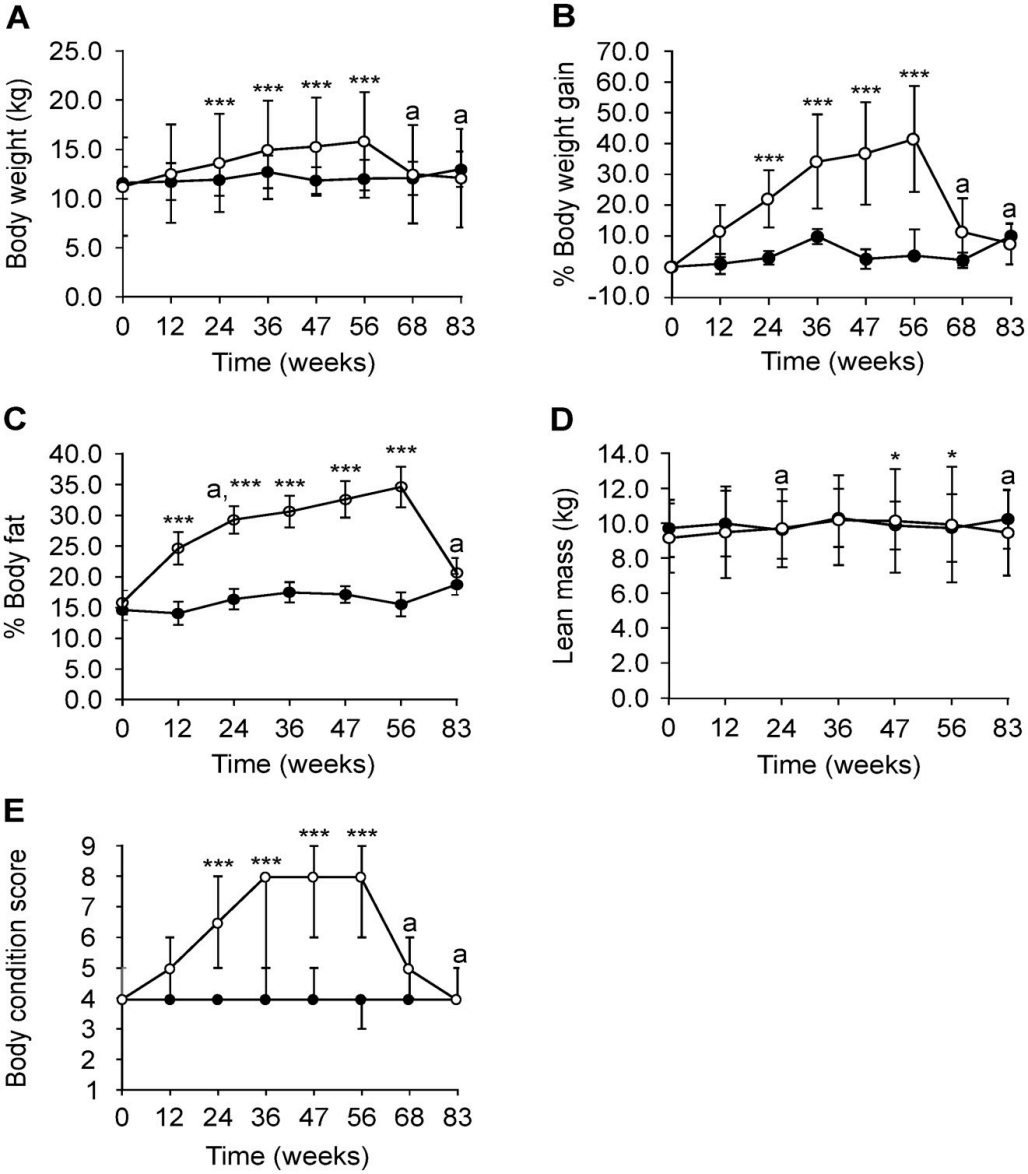
Body characteristics of beagles in the control (closed circles; n = 8) and weight-change (open circles; n = 8) group during the weight-gain phase (week 0–47), weight-stable phase (week 47–56) and weight-loss phase (week 56–83). Body weight (kg) (A), % body weight gain (B), % body fat (C) and body condition score (E) increased significantly from week 12 or week 24 until week 56 in the weight-change group compared to the control group. Lean mass (kg) was significantly increased at week 47 and 56 in the weight-change group. Data is shown as mean ± SD. ^a^Data from one dog missing in the control group. ***P = 0–0.001, **P = 0.001–0.01 and *P = 0.01–0.05 for the group x time interaction, after multiple correction.

After 47 weeks of feeding 1.4 ± 0.5 x maintenance energy requirements (869 ± 156 kJ/kg^0.75^ ideal body weight), three dogs were overweight and five were obese in the weight-change group (BCS 8/9 (6–9)) with a 36.8 ± 16.6% gain in body weight. Body fat increased 16.8 ± 7.7%, which was equivalent to a body fat % of 32.6 ± 5.1%. The values of body weight (week 24: 12.0 ± 1.7 vs. 13.7 ± 2.3 kg, week 36: 12.7 ± 1.7 vs. 15.0 ± 2.6 kg, week 47: 11.9 ± 1.4 vs. 15.3 ± 3.0 kg, week 56: 12.1 ± 2.0 vs. 15.9 ± 3.4 kg; all P < 0.001), body fat % (week 12: 14.1 ± 5.5 vs. 24.7 ± 4.9%, week 24: 16.4 ± 4.6 vs. 29.3 ± 4.7%, week 36: 17.5 ± 5.8 vs. 30.7 ± 7.5%, week 47: 17.2 ± 6.4 vs. 32.6 ± 5.1%, week 56: 15.5 ± 4.8 vs. 34.7 ± 5.2%; all P < 0.001) and BCS (week 24: 4 (4–4) vs. 6.5 (5–7), week 36: 4 (4–5) vs. 8 (5–8), week 47: 4 (4–5) vs. 8 (6–9), week 56: 4 (3–4) vs. 8 (6–9); all P < 0.001) were significantly higher in the weight-change group compared to the control group from week 12 to 56 (Fig 4A–4C, 4E). From week 47 to 56, the weight-change group continued to gain body weight and body fat despite a slight decrease in energy intake (843 ± 127 kJ/kg^0.75^ ideal body weight). Lean mass (kg) also significantly increased at these two time points while the BCS remained stable (week 47: 9.9 ± 1.8 vs. 10.2 ± 2.4 kg, P = 0.018; week 56: 9.8 ± 1.9 vs. 10.0 ± 2.5 kg, P = 0.040) (Fig 4D). During weight-loss (week 68–83), energy intake of the weight-change group was 548 ± 146 kJ/kg^0.75^ ideal body weight. By week 83, the weight-change group lost 23.4 ± 6.8% of body weight and the BCS was 4/9 for all dogs. The body fat % of this group also decreased from week 56 to 83 (-14.0 ± 4.2%). In contrast, the BCS of the control group remained within the ideal range (BCS 4–5) throughout the study, except for one dog at week 56 (with BCS 3). Energy intake throughout the study was 659 ± 156 kJ/kg^0.75^ based on ideal body weight.

### Clinical characteristics and associations within the weight-change group

Results for sBP, serum leptin, serum creatinine, BUN, mGFR, UPC, sCysC and the urinary biomarkers; and for the CEUS parameters of the cortex and medulla of both groups are reported in Table 1, S2 and S3 Tables. uCRP concentrations were below the detection limit of the assay for all samples (5.28 ng/mL). Because of the large number of variables examined in this study, parameters that were most likely influenced by obesity over time were first identified in the weight-change group (Table 2). No significant associations with body characteristics (body fat % and lean mass) were found for sBP, serum creatinine, BUN and uRBP/c. Values of serum creatinine and BUN for all dogs were <1.4 mg/dL and within laboratory reference ranges throughout the study, respectively. Serum leptin and sCysC were significantly and positively correlated with both body fat % and lean mass (P < 0.05). Of the other kidney-related measurements, an increase in body fat % was related to an increase in UPC, uIgG/c and uNGAL/c, and a decrease in mGFR (P < 0.05). In the cortex, intensity-related parameters PE, WiAUC, WoAUC and AUC were mainly negatively correlated with body fat %, while time-related parameters were mainly negatively correlated with lean mass (P < 0.05). Only TTP was negatively correlated with both body fat % and lean mass (P < 0.05). In the medulla, PE, WiR, WiPI and WoR increased as lean mass increased (P < 0.05).

**Table 1:** Systolic blood pressure (sBP), serum leptin, serum creatinine (sCr), blood urea nitrogen (BUN) and glomerular filtration rate (mGFR) measured by exo-iohexol plasma clearance over time in the control group (CG) and the weight-change group (WCG) in dogs.

	Group	Week 0	Week 12	Week 24	Week 36	Week 47	Week 56	Week 68	Week 83
sBP (mm Hg)	CG	153.6 ± 15.9	136.5 ± 10.9	147.3 ± 8.8	131.3 ± 11.5	136.3 ± 11.3	138.5 ± 10	136.2 ± 10.5^a^	131.5 ± 6.2^a^
	WCG	157.0 ± 14.4	141.2 ± 9.0	154.2 ± 8.3	144.7 ± 11.2	148.8 ± 13.5	155.2 ± 13.5	144 ± 8.9	139.5 ± 8.3
Serum leptin (ng/mL)	CG	0.52 ± 0.00	0.52 ± 0.00	0.64 ± 0.34	0.64 ± 0.34	0.52 ± 0.00	0.64 ± 0.34	0.52 ± 0.00^a^	0.79 ± 0.46^a^
	WCG	0.64 ± 0.34	0.99 ± 0.51	1.84 ± 0.70	2.39 ± 1.39^§^	2.66 ± 1.30*	3.15 ± 1.68*	0.99 ± 0.51	0.76 ± 0.44
sCr (mg/dL)	CG	0.63 ± 0.05	0.59 ± 0.07	0.61 ± 0.06	0.63 ± 0.06	0.61 ± 0.06	0.59 ± 0.09	0.64 ± 0.08^a^	0.69 ± 0.10^a^
	WCG	0.63 ± 0.07	0.59 ± 0.11	0.59 ± 0.07	0.61 ± 0.03	0.60 ± 0.07	0.58 ± 0.08	0.64 ± 0.10	0.63 ± 0.09
BUN (mg/dL)	CG	30.7 ± 10.9	28.3 ± 5.2	26.7 ± 5.2	26.7 ± 5.1	26.4 ± 3.2	31.0 ± 11.5	29.5 ± 6.8^a^	31.5 ± 8.8^a^
	WCG	27.4 ± 4.3	30.2 ± 3.1	27.3 ± 4.1	23.0 ± 5.1	26.3 ± 5	26.4 ± 4.2	28.4 ± 10.4	28.0 ± 3.9
mGFR (mL/min/kg)	CG	3.92 ± 0.91	3.78 ± 0.73	3.61 ± 0.76	3.27 ± 0.62	4.15 ± 0.81	4.97 ± 1.02		5.33 ± 1.03^a^
	WCG	3.83 ± 0.58	3.53 ± 0.47	3.33 ± 0.38	2.96 ± 0.84	3.79 ± 0.74	4.13 ± 0.67		5.28 ± 1.17

Data are presented as mean ± SD of 8 beagles per group for all time points. The control group (n = 8) was fed to maintain an ideal body weight throughout the study. The weight-change group (n = 8) was fed to induce obesity (week 0–47), maintain a stable body weight (week 47–56) and to lose weight (week 56–83). mGFR was not measured during week 68.

^a^Data from one dog missing.

*P = 0–0.001 and ^§^P = 0.001–0.01, after multiple correction, for the group x time interaction.

**Table 2:** Association of serum leptin, biomarkers of kidney function and kidney injury and CEUS renal perfusion variables with body fat (BF) percentage and lean mass (kg) within the weight-change group in dogs.

	BF (%)			Lean mass (kg)		
Variables	Estimate	SE	P-value	Estimate	SE	P-value
Serum leptin (ng/mL)	4.86	0.49	**<0.001**	0.25	0.05	**<0.001**
mGFR (mL/min/kg)	-3.85	1.02	**0.002**	-0.14	0.09	0.387
sCysC (mg/L)	166.52	28.31	**<0.001**	13.59	2.72	**<0.001**
UPC	10.37	2.64	**0.003**	0.39	0.24	0.353
uIgG/c (mg/g)	0.07	0.03	**0.035**	0	0	0.936
uNGAL/c (ng/g)	0.53	0.12	**<0.001**	0.03	0.01	0.06
uRBP/c (mg/g)	36.8	15.8	0.1	-0.38	1.6	1
PE	-2.7	0.73	**0.001**	0.07	0.06	0.595
WiAUC	-3.49	0.69	**<0.001**	0	0.06	1
AUC	-3.5	0.68	**<0.001**	-0.01	0.05	1
WoAUC	-3.49	0.67	**<0.001**	-0.02	0.05	1
mTT	-1.95	0.81	**0.048**	-0.01	0.06	1
RT	-1.64	0.83	0.17	-0.22	0.06	**0.001**
TTP	-2.7	0.97	**0.038**	-0.22	0.07	**0.013**
FT	-1.32	0.78	0.289	-0.19	0.06	**0.002**
WiPI	-2.7	0.73	**0.001**	0.07	0.06	0.603
PE	0.15	0.79	1	0.15	0.05	**0.017**
WiR	0.99	0.76	0.539	0.17	0.05	**0.004**
WiPI	0.12	0.79	1	0.15	0.06	**0.016**
WoR	1.13	0.74	0.357	0.14	0.05	**0.018**

The weight-change group (n = 8) was fed to induce obesity (week 0–47), maintain a stable body weight (week 47–56) and to lose weight (week 56–83). Urinary C-reactive protein concentrations were consistently below detection levels. CEUS, contrast-enhanced ultrasound; SE, standard error; mGFR, measured glomerular filtration rate; sCysC, serum cystatin C; UPC, urinary protein:creatinine ratio; uIgG/c, urinary immunoglobulin G:creatinine ratio; uNGAL/c, urinary neutrophil gelatinase-associated lipocalin:creatinine ratio; uRBP/c, urinary retinol-binding protein:creatinine ratio; PE, peak enhancement; WiAUC, wash-in area-under-the curve; AUC, total area-under-the curve; WoAUC, wash-out area-under-the curve; mTT, mean transit time; RT, rise time; TTP, time-to-peak; FT, fall time; WiR, wash-in rate; WiPI, wash-in perfusion index; WoR, wash-out rate. P < 0.05 was considered significant (bold).

### Serum leptin, kidney function and injury, and CEUS renal perfusion variables of the control group and weight-change group over time

Serum leptin concentrations of the weight-change group were significantly higher at week 36 (0.64 ± 0.34 vs. 2.39 ± 1.39 ng/mL, P = 0.002), 47 (0.52 ± 0.00 vs. 2.66 ± 1.30 ng/mL, P < 0.001) and 56 (0.64 ± 0.34 vs. 3.15 ± 1.68 ng/mL, P < 0.001) compared to the control group and decreased after weight-loss (Table 1).

**Fig 5:**
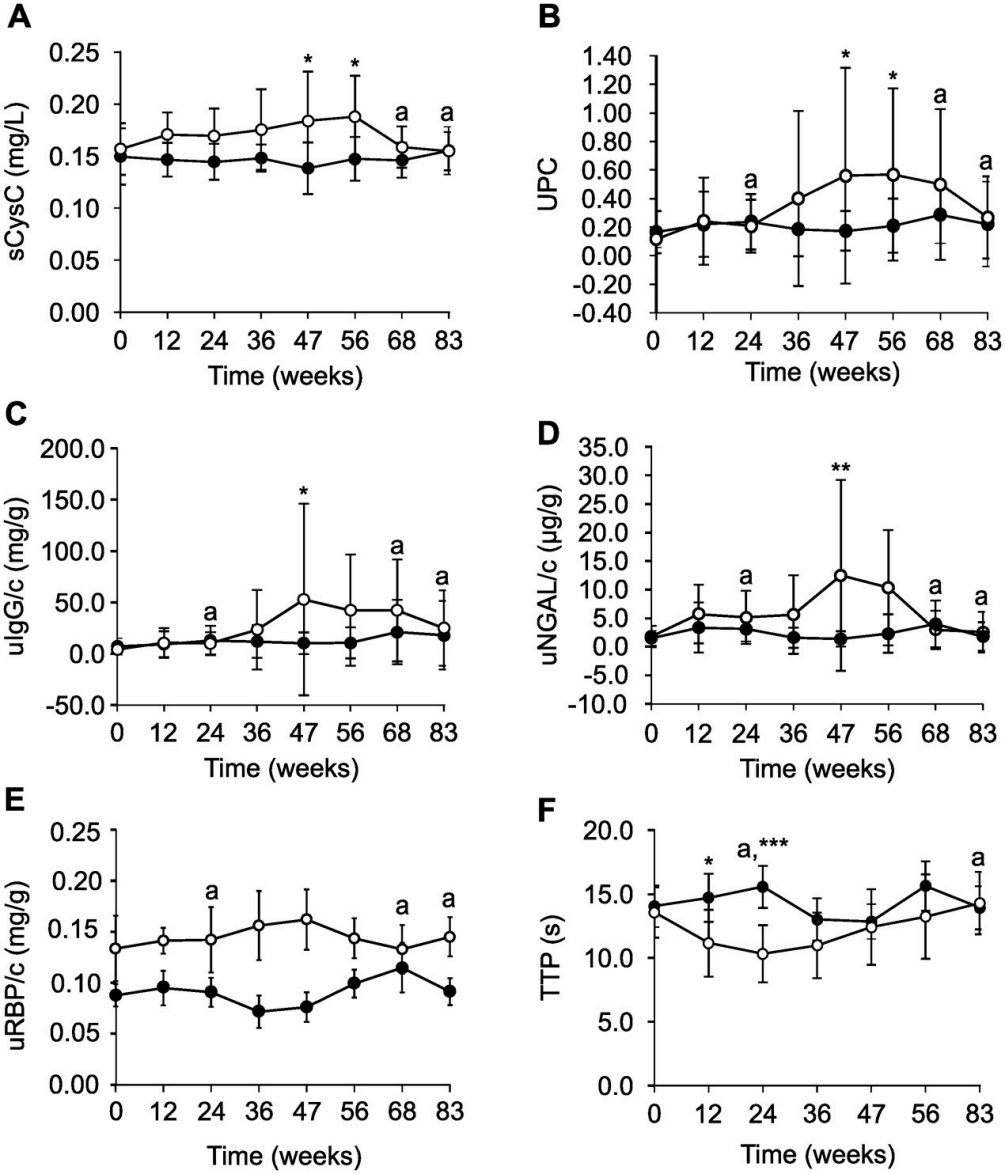
Kidney-related parameters of beagles in the control group (closed circles; n = 8) and in the weight-change group (open circles; n = 8) during the weight-gain phase (week 0–47), weight-stable phase (week 47–56) and weight-loss phase (week 56–83). Serum cystatin C (sCysC, A), a biomarker of kidney function; urinary protein:creatinine (UPC) (B), urinary immunoglobulin G:creatinine (uIgG/c, C), a biomarker of renal glomerular injury; and urinary neutrophil gelatinase-associated lipocalin:creatinine (uNGAL/c, D) a biomarker of renal tubular injury; urinary retinol-binding protein (uRBP/c, E) and time-to-peak (TTP) of the renal cortex, representing blood velocity, measured with contrast-enhanced ultrasound (F) are shown. ^a^Data from one dog missing in the control group. ***P = 0–0.001, **P = 0.001–0.01 and *P = 0.01–0.05 for the group x time interaction, after multiple correction.

No significant differences were found between both groups for exo-iohexol plasma clearance, as marker for mGFR, over time. Both sCysC and UPC levels, however, were significantly higher in the weight-change group than in the control group at week 47 (sCysC: 0.14 ± 0.1 vs. 0.19 ± 0.1 mg/L, P = 0.011; UPC: 0.18 ± 0.14 vs. 0.56 ± 0.76, P = 0.038) and 56 (sCysC: 0.15 ± 0.1 vs. 0.19 ± 0.1 mg/L, P = 0.019; UPC: 0.21 ± 0.19 vs. 0.57 ± 0.61, P = 0.034) (Fig 5A and 5B). At week 83, sCysC and UPC levels of the weight-change group decreased.

The values of uIgG/c and uNGAL/c increased significantly at week 47 in the weight-change group compared to the control group (uIgG/c: 10.3 ± 10.7 vs. 52.9 ± 93.3 mg/g, P = 0.044; uNGAL/c: 1.4 ± 1.4 vs. 12.6 ± 16.8 ng/g, P = 0.006) and decreased from week 56 to 83 (Fig 5C and 5D). Of all CEUS renal perfusion parameters, only cortical TTP was significantly different. It was shorter at week 12 (14.8 ± 5.3 vs. 11.2 ± 2.3, P = 0.018) and 24 (15.6 ± 2.9 vs. 10.4 ± 1.6, P < 0.001) in the weight-change group compared to the control group and gradually increased from week 36 to 83 (Fig 5F).

Two out of eight dogs in the weight-change group had proteinuria at week 47 (UPC 1.67 ± 0.85) and 56 (UPC 1.53 ± 0.25). Only one of both dogs remained proteinuric at week 83 (UPC 0.95). In the control group, from week 12, one dog had persistent but mild proteinuria (UPC 0.62 ± 0.14) without azotemia.

## Discussion

Obesity increases the risk of developing obesity-related glomerulopathy and chronic kidney disease in humans [2,37]. Like chronic kidney disease, diagnosing obesity-related kidney disease at an early stage is challenging as routine measures of kidney function might not be sensitive enough [4]. More sensitive diagnostic techniques to detect kidney injury and changes in function early on in the disease might be useful in monitoring and maybe prevention of obesity-related kidney disease [4]. The aim of this study was to assess when and how changes in kidney function, perfusion and injury occur as dogs gradually became obese and subsequently lost weight. This is the first longitudinal study involving obesity development and subsequent weight-loss in the same subject. Evaluation of the kidney was based on routine and novel markers of kidney function and injury as well as on CEUS as an alternative method to assess renal perfusion in dogs. An alteration in renal perfusion was observed with CEUS early in obesity development, prior to increases in the functional marker sCysC, UPC, and novel biomarkers of glomerular and tubular injury uIgG/c and uNGAL/c, respectively. Moreover, weight-loss reversed these changes. Additionally, serum leptin was significantly elevated just prior to elevations in UPC, uIgG/c and uNGAL/c, which might have played a role in the observed elevations. Leptin stimulates glomerular endothelial cells proliferation and transforming growth factor-β1 expression/secretion, a profibrinogenic cytokine and contributor to glomerulosclerosis development [38].

Cortical TTP was significantly shorter in the weight-change group at the beginning of the weight-gain phase, indicating that the cortex took less time to completely perfuse compared to the control group. Medullary TTP was not significantly different between the groups and might be due large with-in dog variation which could be caused by respiratory movement and the presence of interlobar arteries [25,39,40]. Interestingly, at that time point, the weight-change group dogs still had an ideal body weight and condition [24], suggesting that even with slight weight gain (i.e., 11–22%), a physiological response might have occurred paving the way to kidney damage as suggested by increases uIgG/c and uNGAL/c. With 50% weight-gain in a porcine model of metabolic syndrome, cortical perfusion (measured by multidetector computed tomography) also increases, accompanied by proliferation of 20- to 40-μm cortical microvessels and expression of angiogenic activity proteins, e.g., vascular endothelial growth factor (VEGF) [41]. In contrast, in a murine obesity-related glomerulopathy model, mice with a 200% increase in body weight have longer cortical TTP, detected by CEUS, with concurrent capillary density reduction compared to control mice [15]. These studies suggest that when weight-gain is minimal to 50%, renal cortical perfusion increases, possibly due to angiogenesis. However, newly-formed microvessels might not function properly yet and instead attract inflammatory cytokines that partake in initiating renal injury [41]. Furthermore, overexpression of VEGF is strongly associated with development of glomerular endothelial lesions in obesity-related glomerulopathy patients [42]. Extreme weight-gain can exacerbate hemodynamic, metabolic and inflammatory changes leading to microvascular rarefaction and kidney injury [12]. The latter process might have occurred in the weight-change group as urinary biomarkers of renal injury increased when body weight gain was maximal even though cortical TTP was no longer significantly different compared to the control group.

Renal TTP, however, can also be influenced by other factors, e.g., heart rate and cardiac output [43]. In the canine model of obesity-induced hypertension, 10% weight-gain increases heart rate, cardiac output, and blood pressure [44]. However, weight-gain is often induced by feeding a high amount of fat in canine models of obesity [16–18]. In fact, dogs already show increased heart rate on the first day of a diet supplemented with beef fat [45]. Therefore, TTP could also be affected by diet. In the current study, absolute dietary fat intake increased with food intake in the weight-change group. However, the diet was the same for both groups and thus the macronutrient ratio remained the same and was not extreme compared to other studies [16–18]. Furthermore, increased cardiac output in obesity is more strongly correlated with fat-free body mass than with fat mass [46]. In the weight-change group, lean mass increased significantly only at the end of the weight-gain phase so cardiac output might have not have been the main contributor to the change in TTP. Nevertheless, although sBP did not significantly differ between the two groups, interpretation of TTP must be made with caution as heart rate and cardiac output were not measured.

In contrast to many studies involving obesity-related renal disease, mGFR and sBP were not significantly different between the two groups [16,47]. VEGF might be responsible for increasing renal blood flow without a concomitant increase in GFR. Not only is VEGF an angiogenic factor, it is also a vasodilator via nitric oxide synthesis [48]. In a study of the isolated perfused rat kidney, VEGF increased the renal blood flow without a change in glomerular filtration rate and permselectivity [49]. Furthermore, the protein excretion rate of rats infused with VEGF into the renal artery was not influenced [49]. Although VEGF is also involved in the induction of fenestration in endothelial cells, the authors of the study suggest that fenestrae expansion of the glomerular endothelia were already maximal and could explain the observations seen [49]. Moreover, in obese Zucker rats, renal cortical vascularization is increased at an early stage and accompanied by higher levels of VEGF, which might be a compensatory mechanism to maintain renal perfusion [50]. However, a more likely explanation for our results is that the weight-gain in the weight-change group was either not pronounced or chronic enough to induce changes in mGFR. Renal function biomarker sCysC, however, was significantly increased with weight-gain and decreased with weight-loss. The latter is consistent with a weight-loss study in obese pet dogs [51]. Although an increase in sCysC suggests a decrease in kidney function [52], it must be interpreted with caution. In obese humans, concentrations of sCysC and mRNA expression in adipose tissue are consistently higher, independent of estimated GFR [6]. Moreover, sCysC is significantly associated with body fat % [53], like in our study. Although this biomarker seems to be unaffected by muscle mass unlike serum creatinine [53], our study found a significant association with lean mass, similar to another study [54].

Our study confirmed that obesity increases urinary protein excretion as body fat % was also significantly associated with UPC [2]. However, it is also in contrast to a pet dog study in which UPC values between lean and obese animals did not significantly differ [41,55]. The limited number of obese and severely obese pet dogs recruited might have been a factor in the discrepancy. Furthermore, although UPC also decreased with weight-loss, similar to previous canine and human studies [3,51], the extent to which the kidneys experienced injury remains to be documented. The two proteinuric weight-change group dogs during the study were still proteinuric approximately 6 months after the study ended despite a lean body weight and no other relevant clinical abnormalities.

While UPC is useful in assessing renal function in combination with other routine biomarkers, it lacks specificity regarding identification of both the cause and location of renal injury [56]. Studies using site-specific biomarkers of renal injury in obesity are limited. In the current study, two glomerular and two tubular biomarkers of renal injury were evaluated. Unexpectedly, only one of each type (uIgG/c and uNGAL/c, respectively) was significantly increased at the end of the weight-gain phase in the weight-change group. The severity of the induced kidney lesions might partly explain this finding. For example, in patients with rheumatoid arthritis and renal amyloidosis, uCRP is only measurable in combination with heavy proteinuria (i.e., >3 g/24 h) [57]. As uCRP was undetectable in the samples of our study, uIgG/c might be a more sensitive biomarker for early or mild glomerular injury. If used in combination with other urinary biomarkers, uCRP/c might still be useful in determining the severity of glomerular damage.

uNGAL/c significantly increased in the weight-change group at the end of the weight-gain phase. However, in other studies uNGAL/c values are inconsistent in obese subjects, reflecting discrepancies in the type of lesions found (i.e., glomerular or tubular only or both) [58–63]. The combined increase in UPC, uIgG/c and uNGAL/c suggests a degree of permeability defect and tubular injury during the weight-gain phase. However, uRBP/c, the other tubular biomarker, did not change significantly over time. For uRBP levels to increase, an impairment in the glomerular filtration barrier causing competition for the reabsorption of proteins, massive production of low-molecular weight proteins that surpasses the capacity of proximal tubular reabsorption, or impairment of the reabsorption machinery has to occur [64]. In this study, it might be possible that the degree of the permeability defect was too low in the weight-change dogs to have caused competition for reabsorption and that the proximal tubular cells still had the capacity to reabsorb protein. However, the levels of high-molecular weight proteins, such as uIgG, was probably high enough to cause expression and detectable increases of uNGAL, as exposure of the proximal tubular epithelial cells to such proteins stimulates inflammatory responses and apoptosis [65–67]. Damaged tubular cells respond by actively producing uNGAL as a defense mechanism to counteract intracellular oxidative stress and complement- induced apoptosis [66].

The current study has some limitations. First, because of its invasiveness, no renal biopsies were performed. Histopathologic examination of biopsies might confirm or dispute the observed urinary biomarkers changes. Similarly, tissue studies assessing the renal microvasculature and expression of VEGF could also have been useful. The main reasons renal biopsies were not performed were 1) ethical considerations, as the most common complication is severe hemorrhage, and complications are more likely to occur in dogs between 4 to 7 years or greater than 9 years, like the majority of the dogs in our study [68]; and 2) as this was a longitudinal study, there is a possible risk that persistent iatrogenic lesions in the kidney due repeated biopsies could occur [69] and could confound the findings of the kidney injury markers. In a previous study, worsening histologic changes, such as glomerulosclerosis and interstitial fibrosis after repeated biopsies in dogs (8.4 to 11 years) were found [70]. However, it is not clear whether the changes could be attributed to the biopsies or to age-related lesions or progression of existing lesions. Furthermore, the scope of the study was to assess whether non-invasive techniques such as CEUS and novel renal biomarkers could provide extra information beyond that of routine measures on kidney function and injury potentially induced by obesity.

Second, a gold standard for measuring renal blood flow was not used in this study. Although measuring para-amino hippuric acid clearance is considered the gold standard to estimate renal plasma flow, is moderately correlated to CEUS in humans (*r* = 0.69) [71]. Furthermore, currently, there is no gold standard for assessing microperfusion, as para-amino hippuric acid clearance is a measure of global renal perfusion [72].

Third, because the study lasted 1.5 years, functional and structural changes of the kidney, e.g., proteinuria and glomerulosclerosis, as a consequence of aging must also be considered [73]. However, the CG was age-matched, so age-related changes are expected to occur equally in both groups.

Fourth, CEUS is vulnerable to variation caused by differences in e.g., scanner settings, the contrast agent used, and patient-related factors [43]. Part of this variation can be overcome by using a standardized protocol, as was done for this study, but patient-related factors are more difficult to control [43]. Intra-individual variation of CEUS perfusion parameters using a standardized protocol, with an interval between 9 and 27 weeks, in dogs was very similar to the variation found in cats that were examined with 7-day intervals [25,39]. Nevertheless, variation for varies widely for the perfusion parameters with TTP of the cortex having the least amount of variation across time (coefficient of variation of 15%) [25].

Fifth, differences in sodium intake between the two groups could have affected hemodynamics. As obesity was induced by increasing the amount of the same feed, sodium intake was most likely higher during the weight gain phase of the weigh-change group (S2 Table). While an increase in blood pressure, volume and flow after receiving a diet containing high amounts of sodium (4 g/kg body weight) have been seen in an experimental study using dogs, the sodium content was considerably higher than what the dogs received in our study [74]. Dogs are able to tolerate a wide range of sodium concentrations in the diet as long as access to water remains available [75].

Although the study had random, age-matched groups, there could have been other parameters might have had an influence on the outcome parameters. However, because of the small sample size, it is difficult to fully explore them. Larger studies in the future would be helpful in detecting other confounding factors.

Lastly, this study used diet-induced obese dogs. Because the duration that dogs remained obese was relatively short, cut-off points cannot be made and the results cannot be extrapolated to humans. Furthermore, it is unknown if CEUS and the selected novel renal biomarkers are also useful in human obesity. However, the potential exists as dogs are good models of obesity and obesity-related glomerulopathy in humans due to similarities in fat accumulation, genetic background and comorbidities [76,77]. Furthermore, previous experiments have shown that demonstrate that when dogs are used as a model for obesity-induced hypertension or obesity-induced renal disease, hemodynamic changes closely mimic those of obese humans, such as an increase in arterial blood pressure, GFR, and renal blood flow [16,23,76]. The reason our study did not observe increased blood pressure and GFR during the weight-gain phase is unclear. The major differences compared to the previous studies are that those studies induce weight-gain at a faster rate and the dogs are fed a diet high in fat or supplemented with fat.

In conclusion, CEUS detected alterations in renal perfusion early in obesity development in dogs, without hyperfiltration and prior to increases in biomarker concentrations of kidney function (i.e., sCysC) and injury (i.e., uIgG/c and uNGAL/c); and is reversed with weight-loss. The results of this study can serve as a starting point for studies in human obesity-related kidney disease and for the development of more sensitive and non-invasive techniques to detect early alterations in kidney function, perfusion and injury. Combining CEUS and urinary biomarkers to monitor renal function and injury might be more sensitive in identifying and monitoring individuals at-risk for obesity-related glomerulopathy than traditional techniques alone. Future studies should investigate the clinical applicability of CEUS and the selected urinary kidney injury and serum functional biomarkers in overweight and obese individuals.

## Supporting information

S1 Video Contrast-enhanced ultrasonography of the left canine kidney in the longitudinal plane. This recording demonstrates the first 20 seconds after a bolus injection of sulfur hexafluoride-filled microbubbles (SonoVue^®^, Bracco Diagnostics Inc., Milan, Italy).
(MP4)

S1 Table Analyzed nutrient composition of the commercial canine adult maintenance diet*. NFE, nitrogen- free extract; TDF, total dietary fiber; IDF, insoluble dietary fiber; SDF, soluble dietary fiber; ME, metabolizable energy *Diet ingredients, Virbac Veterinary™ HPM Adult Large and Medium: Dehydrated pork and poultry protein, rice (min. 7%), whole pea, animal fat, hydrolyzed animal protein, potato starch (min. 4%), lignocellulose, linseed field bean hulls, mineral salts, beet pulp, fructo-oligosaccharides, psyllium fiber, chitosan, pasteurized *Lactobacillus acidophilus*, chondroitin sulfate. ^§^Calculated as 100 − (crude protein + crude fat + crude ash + crude fiber) ^†^Estimated using a four-step calculation [27].
(DOCX)

S2 Table Body characteristics and kidney-related measurements over time of dogs in the control group (CG) and the weight-change group (WCG). Data are presented as mean ± SD of 8 beagles per group for all time points. Beagles in the control group (n = 8) were fed to maintain an ideal body weight throughout the study. The weight-change group (n = 8) was fed to develop obesity (week 0–47), to maintain a stable body weight (week 47–56) and to lose weight (week 56–83). ^a^Data from one dog missing. ^b^Fat mass and sodium intake were not included in the statistical analysis. BW, body weight; UPC, urine protein:creatinine ratio; sCysC, serum cystatin C (sCysC); uRBP/c, urinary retinol-binding protein:creatinine ratio; uNGAL/c, urinary neutrophil gelatinase-associated lipocalin:creatinine; uIgG/c, urinary immunoglobulin G:creatinine. *P = 0–0.001, ^§^P = 0.001–0.01 and ^†^P = 0.01–0.05, after multiple correction, for the group x time interaction.
(DOCX)

S3 Table CEUS renal perfusion variables from the left kidney over time in dogs from the control group (CG) and the WG group (WCG). Data are presented as mean ± SD of 8 beagles per group for all time points. Beagles in the control group (n = 8) were fed to maintain an ideal body weight throughout the study. The weight-change group (n = 8) was fed to develop obesity (week 0–47), to maintain a stable body weight (week 47–56) and to lose weight (week 56–83). ^a^Data from one dog missing at week 24 and week 83. PE, peak enhancement; WiAUC, wash-in area under the curve; AUC, total area under the curve; WoAUC, wash-out area under the curve; mTT, mean transit time; RT, rise time; TTP, time-to-peak; FT, fall time; WiR, wash-in rate; WiPI, wash-in perfusion index; WoR, wash-out rate. *P = 0–0.001, ^§^P = 0.001–0.01 and ^†^P = 0.01–0.05, after multiple correction, for the group x time interaction.
(DOCX)
