# Supplementary material for: Weight-gain induced changes in renal perfusion assessed by contrast-enhanced ultrasound precede increases in urinary protein excretion suggestive of glomerular and tubular injury and normalize after weight-loss in dogs
Source: PLoS One. 2020 Apr 21;15(4):e0231662. doi: 10.1371/journal.pone.0231662 (PMC7173781; doi:10.1371/journal.pone.0231662)
Supplement: S1 Table — NFE, nitrogen- free extract; TDF, total dietary fiber; IDF, insoluble dietary fiber; SDF, soluble dietary fiber; ME, metabolizable energy *Diet ingredients, Virbac Veterinary™ HPM Adult Large and Medium: Dehydrated pork and poultry protein, rice (min. 7%), whole pea, animal fat, hydrolyzed animal protein, potato starch (min. 4%), lignocellulose, linseed field bean hulls, mineral salts, beet pulp, fructo-oligosaccharides, psyllium fiber, chitosan, pasteurized Lactobacillus acidophilus, chondroitin sulfate. §Calculated as 100 − (crude protein + crude fat + crude ash + crude fiber) †Estimated using a four-step calculation [27]. (DOCX) [file pone.0231662.s002.docx]

## S1 Table. Analyzed nutrient composition of the commercial canine adult maintenance diet^*^

| **Nutrients** | **% on dry matter basis** | |
| --- | --- | --- |
| Crude fat |  | 18.2 |
| Crude protein |  | 36.7 |
| Crude ash |  | 7.8 |
| Crude fiber |  | 3.3 |
| NFE^§^ |  | 34.1 |
| TDF |  | 28.2 |
| IDF |  | 23.6 |
| SDF |  | 4.6 |
| ME (kJ/100 g as fed)^†^ |  | 1769 |

NFE, nitrogen- free extract; TDF, total dietary fiber; IDF, insoluble dietary fiber; SDF, soluble dietary fiber; ME, metabolizable energy

^*^Diet ingredients, Virbac Veterinary™ HPM Adult Large and Medium: Dehydrated pork and poultry protein, rice (min. 7 %), whole pea, animal fat, hydrolyzed animal protein, potato starch (min. 4 %), lignocellulose, linseed field bean hulls, mineral salts, beet pulp, fructo-oligosaccharides, psyllium fiber, chitosan, pasteurized *Lactobacillus acidophilus*, chondroitin sulfate.

^§^Calculated as 100 - (crude protein + crude fat + crude ash + crude fiber)

^†^Estimated using a four-step calculation^24^
