# Supplementary material for: Weight-gain induced changes in renal perfusion assessed by contrast-enhanced ultrasound precede increases in urinary protein excretion suggestive of glomerular and tubular injury and normalize after weight-loss in dogs
Source: PLoS One. 2020 Apr 21;15(4):e0231662. doi: 10.1371/journal.pone.0231662 (PMC7173781; doi:10.1371/journal.pone.0231662)
Supplement: S2 Table — Data are presented as mean ± SD of 8 beagles per group for all time points. Beagles in the control group (n = 8) were fed to maintain an ideal body weight throughout the study. The weight-change group (n = 8) was fed to develop obesity (week 0–47), to maintain a stable body weight (week 47–56) and to lose weight (week 56–83). aData from one dog missing. bFat mass and sodium intake were not included in the statistical analysis. BW, body weight; UPC, urine protein:creatinine ratio; sCysC, serum cystatin C (sCysC); uRBP/c, urinary retinol-binding protein:creatinine ratio; uNGAL/c, urinary neutrophil gelatinase-associated lipocalin:creatinine; uIgG/c, urinary immunoglobulin G:creatinine. *P = 0–0.001, §P = 0.001–0.01 and †P = 0.01–0.05, after multiple correction, for the group x time interaction. (DOCX) [file pone.0231662.s003.docx]

| **S2 Table. Body characteristics and kidney-related measurements over time of dogs in the control group (CG) and the weight-change group (WCG)** | | | | | | | | | |
| --- | --- | --- | --- | --- | --- | --- | --- | --- | --- |
|  | Group | Week 0 | Week 12 | Week 24 | Week 36 | Week 47 | Week 56 | Week 68 | Week 83 |
| *Body characteristics* | | | | | | | | | |
| Body weight (kg) | CG | 11.6 ± 1.7 | 11.8 ± 1.9 | 12.0 ± 1.7 | 12.7 ± 1.7 | 11.9 ± 1.4 | 12.1 ± 2.0 | 12.1 ± 1.7^a^ | 13.0 ± 1.8^a^ |
|  | WCG | 11.2 ± 2.0 | 12.6 ± 2.7 | 13.7 ± 2.3^*^ | 15.0 ± 2.6^*^ | 15.3 ± 3.0^*^ | 15.9 ± 3.4^*^ | 12.5 ± 2.5 | 12.1 ± 2.5 |
| % overweight | CG | 0.0 ± 0.0 | 1.0 ± 3.3 | 3.0 ± 2.2 | 9.9 ± 2.5 | 2.6 ± 3.2 | 3.6 ± 4.3 | 2.2 ± 2.5^a^ | 10.1 ± 3.9^a^ |
|  | WCG | 0.0 ± 0.0 | 11.6 ± 8.5 | 22.1 ± 9.3^*^ | 34.3 ± 15.3^*^ | 36.9 ± 16.7^*^ | 41.6 ± 17.2^*^ | 11.4 ± 10.9 | 7.5 ± 6.7 |
| Body condition score | CG | 4 (4 - 4) | 4 (4 - 4) | 4 (4 - 4) | 4 (4 - 5) | 4 (4 - 5) | 4 (3 - 4) | 4 (4 - 4)^a^ | 4 (4 - 5)^a^ |
|  | WCG | 4 (4 - 5) | 5 (4 - 6) | 6.5 (5 - 7) ^*^ | 8 (5 - 8) ^*^ | 8 (6 - 9) ^*^ | 8 (6 - 9) ^*^ | 5 (4 - 6) | 4 (4 - 4) |
| Body fat (%) | CG | 14.6 ± 6.1 | 14.1 ± 5.5 | 16.4 ± 4.6^a^ | 17.5 ± 5.8 | 17.2 ± 6.4 | 15.5 ± 4.8 | - | 18.8 ± 5.6^a^ |
|  | WCG | 15.8 ± 7.1 | 24.7 ± 4.9^*^ | 29.3 ± 4.7^*^ | 30.7 ± 7.5^*^ | 32.6 ± 5.1^*^ | 34.7 ± 5.2^*^ | - | 20.7 ± 2.1 |
| Lean mass (kg) | CG | 9.8 ± 1.9 | 10.0 ± 2.1 | 9.7 ± 1.6^a^ | 10.4 ± 2.0 | 9.9 ± 1.8 | 9.8 ± 1.9 | - | 10.3 ± 1.6^a^ |
|  | WCG | 9.2 ± 2.0 | 9.6 ± 2.5 | 9.8 ± 2.4 | 10.2 ± 2.5 | 10.2 ± 2.4^†^ | 10.0 ± 2.5^†^ | - | 9.5 ± 2.3 |
| Fat mass (kg)^b^ | CG | 1.7 ± 0.7 | 1.6 ± 0.6 | 1.9 ± 0.6^a^ | 2.2 ± 0.6 | 2.0 ± 0.7 | 1.8 ± 0.5 | - | 2.4 ± 0.8^a^ |
|  | WCG | 1.8 ± 0.9 | 3.1 ± 0.7 | 4.0 ± 0.6 | 4.5 ± 1.3 | 5.0 ± 1.2 | 5.3 ± 1.3 | - | 2.5 ± 0.4 |
| Energy intake (kJ/kg iBW^0.75^) | CG | 687 ± 216 | 661 ± 140 | 687 ± 108 | 638 ± 142 | 614 ± 196 | 633 ± 164 | 690 ± 110^a^ | 669 ± 197^a^ |
|  | WCG | 591 ± 186 | 812 ± 119^*^ | 864 ± 136^*^ | 843 ± 149^*^ | 960 ± 202^*^ | 843 ± 128^*^ | 529 ± 111 | 567 ± 180 |
| Sodium intake (mg/kg iBW^0.75^) ^b^ | CG | 258 ± 81 | 248 ± 53 | 258 ± 41 | 240 ± 54 | 231 ± 74 | 238 ± 62 | 259 ± 42 | 251 ± 74 |
|  | WCG | 222 ± 70 | 305 ± 45 | 325 ± 51 | 317 ± 56 | 361 ± 76 | 317 ± 48 | 199 ± 42 | 213 ± 68 |
| *Kidney-related measurements* | | | | | | | | | |
| sCysC (mg/L) | CG | 0.15 ± 0.1 | 0.15 ± 0.1 | 0.15 ± 0.1 | 0.15 ± 0.1 | 0.14 ± 0.1 | 0.15 ± 0.1 | 0.15 ± 0.1^a^ | 0.16 ± 0.1^a^ |
|  | WCG | 0.16 ± 0.1 | 0.18 ± 0.1 | 0.17 ± 0.1 | 0.18 ± 0.1 | 0.19 ± 0.1^†^ | 0.19 ± 0.1^†^ | 0.16 ± 0.1 | 0.16 ± 0.1 |
| UPC | CG | 0.17 ± 0.15 | 0.22 ± 0.23 | 0.24 ± 0.20^a^ | 0.19 ± 0.19 | 0.18 ± 0.14 | 0.21 ± 0.19 | 0.29 ± 0.20^a^ | 0.23 ± 0.30^a^ |
|  | WCG | 0.12 ± 0.06 | 0.25 ± 0.31 | 0.21 ± 0.19 | 0.40 ± 0.62 | 0.56 ± 0.76^†^ | 0.57 ± 0.61^†^ | 0.50 ± 0.53 | 0.27 ± 0.29 |
| uRBP/c (mg/g) | CG | 0.09 ± 0.04 | 0.10 ± 0.05 | 0.10 ± 0.04^a^ | 0.08 ± 0.05 | 0.08 ± 0.05 | 0.11 ± 0.04 | 0.12 ± 0.07^a^ | 0.10 ± 0.04^a^ |
|  | WCG | 0.14 ± 0.10 | 0.15 ± 0.04 | 0.15 ± 0.10 | 0.16 ± 0.10 | 0.17 ± 0.09 | 0.15 ± 0.06 | 0.14 ± 0.07 | 0.15 ± 0.06 |
| uNGAL/c (ng/g) | CG | 1.6 ± 1.4 | 3.4 ± 4.4 | 3.2 ± 2.2^a^ | 1.6 ± 1.8 | 1.4 ± 1.4 | 2.4 ± 3.4 | 4.1 ± 4.1^a^ | 1.9 ± 2.5^a^ |
|  | WCG | 1.9 ± 1.9 | 5.8 ± 5.2 | 5.2 ± 4.7 | 5.7 ± 6.9 | 12.6 ± 16.8^§^ | 10.4 ± 10.1 | 3.0 ± 3.4 | 2.6 ± 3.6 |
| uIgG/c (mg/g) | CG | 7.1 ± 8.1 | 9.4 ± 12.6 | 13.0 ± 14.3^a^ | 12.0 ± 16.0 | 10.3 ± 10.7 | 10.7 ± 15.2 | 21.2 ± 31.5^a^ | 18.1 ± 33.4^a^ |
|  | WCG | 4.3 ± 3.7 | 10.5 ± 14.5 | 10.2 ± 10.9 | 23.5 ± 38.8 | 52.9 ± 93.3^†^ | 42.5 ± 54.2 | 42.3 ± 49.7 | 25.1 ± 36.8 |
| Data are presented as mean ± SD of 8 beagles per group for all time points. Beagles in the control group (n = 8) were fed to maintain an ideal body weight throughout the study. The weight-change group (n = 8) was fed to develop obesity (week 0 – 47), to maintain a stable body weight (week 47 – 56) and to lose weight (week 56 -83). ^a^Data from one dog missing. ^b^Fat mass and sodium intake were not included in the statistical analysis. BW, body weight; UPC, urine protein:creatinine ratio; sCysC, serum cystatin C (sCysC); uRBP/c, urinary retinol-binding protein:creatinine ratio; uNGAL/c, urinary neutrophil gelatinase-associated lipocalin:creatinine; uIgG/c, urinary immunoglobulin G:creatinine. ^*^P = 0 - 0.001, ^§^P = 0.001 - 0.01 and ^†^P = 0.01 - 0.05, after multiple correction, for the group x time interaction. | | | | | | | | | |
