# Supplementary material for: Weight-gain induced changes in renal perfusion assessed by contrast-enhanced ultrasound precede increases in urinary protein excretion suggestive of glomerular and tubular injury and normalize after weight-loss in dogs
Source: PLoS One. 2020 Apr 21;15(4):e0231662. doi: 10.1371/journal.pone.0231662 (PMC7173781; doi:10.1371/journal.pone.0231662)
Supplement: S3 Table — Data are presented as mean ± SD of 8 beagles per group for all time points. Beagles in the control group (n = 8) were fed to maintain an ideal body weight throughout the study. The weight-change group (n = 8) was fed to develop obesity (week 0–47), to maintain a stable body weight (week 47–56) and to lose weight (week 56–83). aData from one dog missing at week 24 and week 83. PE, peak enhancement; WiAUC, wash-in area under the curve; AUC, total area under the curve; WoAUC, wash-out area under the curve; mTT, mean transit time; RT, rise time; TTP, time-to-peak; FT, fall time; WiR, wash-in rate; WiPI, wash-in perfusion index; WoR, wash-out rate. *P = 0–0.001, §P = 0.001–0.01 and †P = 0.01–0.05, after multiple correction, for the group x time interaction. (DOCX) [file pone.0231662.s004.docx]

| **S3 Table. CEUS renal perfusion variables from the left kidney over time in dogs from the control group (CG) and the WG group (WCG)** | | | | | | | | |
| --- | --- | --- | --- | --- | --- | --- | --- | --- |
|  | Group | Week 0 | Week 12 | Week 24^a^ | Week 36 | Week 47 | Week 56 | Week 83^a^ |
| *Cortex* |  |  |  |  |  |  |  |  |
| PE | CG | 2173 ± 658 | 2198 ± 556 | 1527 ± 612 | 1921 ± 1031 | 1995 ± 763 | 786 ± 229 | 2582 ± 1075 |
|  | WCG | 2584 ± 1414 | 2008 ± 995 | 1566 ± 533 | 2042 ± 775 | 2654 ± 987 | 946 ± 439 | 2639 ± 792 |
| WiAUC | CG | 6600 ± 2620 | 6101 ± 2294 | 4309 ± 1588 | 4432 ± 1141 | 4934 ± 1499 | 2330 ± 1003 | 6506 ± 1827 |
|  | WCG | 6914 ± 2331 | 4666 ± 2116 | 3442 ± 1479 | 4747 ± 1725 | 7059 ± 3045 | 2560 ± 1056 | 6618 ± 865 |
| AUC | CG | 15319 ± 6191 | 13821 ± 5290 | 9766 ± 3729 | 10070 ± 2390 | 11294 ± 3499 | 5348 ± 2569 | 14800 ± 3836 |
|  | WCG | 15905 ± 4880 | 10770 ± 4804 | 7945 ± 3471 | 10990 ± 3764 | 16252 ± 6670 | 5956 ± 2380 | 14993 ± 1702 |
| WoAUC | CG | 8720 ± 3580 | 7716 ± 2997 | 5450 ± 2136 | 5643 ± 1262 | 6364 ± 2019 | 3019 ± 1571 | 8294 ± 2059 |
|  | WCG | 8997 ± 2557 | 6104 ± 2689 | 4505 ± 1997 | 6241 ± 2032 | 9215 ± 3650 | 3397 ± 1341 | 8375 ± 878 |
| mTT | CG | 35.9 ± 4.8 | 31.2 ± 9.6 | 30.2 ± 9.4 | 28.3 ± 10.1 | 31.9 ± 8.9 | 26.4 ± 12.8 | 33.5 ± 13.5 |
|  | WCG | 37.3 ± 10.0 | 38.9 ± 12.0 | 28.0 ± 9.6 | 44.4 ± 29.7 | 39.0 ± 15.9 | 27.1 ± 8.1 | 28.9 ± 9.5 |
| RT | CG | 4.9 ± 0.9 | 4.7 ± 2.1 | 4.9 ± 1.3 | 4.3 ± 1.3 | 4.3 ± 0.7 | 4.9 ± 1.9 | 4.4 ± 0.9 |
|  | WCG | 4.8 ± 1.1 | 4.0 ± 0.4 | 3.6 ± 0.3 | 3.9 ± 0.4 | 4.3 ± 0.3 | 4.6 ± 0.4 | 4.4 ± 0.4 |
| TTP | CG | 14.1 ± 3.0 | 14.8 ± 5.3 | 15.6 ± 2.9 | 13.1 ± 2.4 | 12.9 ± 1.7 | 15.7 ± 2.7 | 14.0 ± 2.5 |
|  | WCG | 13.6 ± 4.1 | 11.2 ± 2.3^†^ | 10.4 ± 1.6^*^ | 11.0 ± 3.8 | 12.5 ± 2.7 | 13.3 ± 1.4 | 14.3 ± 3.1 |
| FT | CG | 6.7 ± 1.4 | 6.2 ± 2.8 | 6.4 ± 1.8 | 5.8 ± 2.0 | 5.7 ± 1.1 | 6.6 ± 3.3 | 5.8 ± 1.6 |
|  | WCG | 6.6 ± 2.5 | 5.4 ± 1.4 | 4.9 ± 1.2 | 5.4 ± 1.5 | 5.9 ± 1.2 | 6.4 ± 1.9 | 5.7 ± 1.5 |
| WiR | CG | 580 ± 164 | 675 ± 237 | 443 ± 231 | 695 ± 547 | 645 ± 319 | 223 ± 89 | 823 ± 475 |
|  | WCG | 824 ± 677 | 699 ± 376 | 582 ± 213 | 744 ± 439 | 802 ± 264 | 284 ± 160 | 869 ± 501 |
| WiPI | CG | 1330 ± 404 | 1339 ± 338 | 932 ± 374 | 1172 ± 625 | 1217 ± 463 | 479 ± 139 | 1576 ± 652 |
|  | WCG | 1578 ± 850 | 1228 ± 607 | 958 ± 326 | 1247 ± 465 | 1624 ± 598 | 578 ± 268 | 1607 ± 478 |
| WoR | CG | 384 ± 105 | 477 ± 179 | 313 ± 169 | 497 ± 417 | 446 ± 246 | 159 ± 68 | 592 ± 388 |
|  | WCG | 572 ± 554 | 463 ± 252 | 392 ± 155 | 495 ± 334 | 538 ± 210 | 195 ± 118 | 624 ± 393 |
|  |  |  |  |  |  |  |  |  |
| *Medulla* |  |  |  |  |  |  |  |  |
| PE | CG | 74 ± 58 | 105 ± 83 | 91 ± 94 | 76 ± 33 | 70 ± 46 | 34 ± 18 | 94 ± 56 |
|  | WCG | 107 ± 72 | 101 ± 50 | 103 ± 74 | 119 ± 32 | 189 ± 157 | 56 ± 28 | 156 ± 102 |
| WiAUC | CG | 975 ± 800 | 1000 ± 567 | 798 ± 608 | 810 ± 466 | 614 ± 361 | 384 ± 252 | 1140 ± 441 |
|  | WCG | 1207 ± 640 | 1358 ± 618 | 893 ± 723 | 1444 ± 432 | 1696 ± 1573 | 694 ± 320 | 1597 ± 1140 |
| AUC | CG | 975 ± 800 | 1000 ± 567 | 798 ± 608 | 810 ± 466 | 614 ± 361 | 384 ± 252 | 1140 ± 441 |
|  | WCG | 1207 ± 640 | 1358 ± 618 | 893 ± 723 | 1444 ± 432 | 1696 ± 1573 | 694 ± 320 | 1597 ± 1140 |
| WoAUC | CG | 1763 ± 1320 | 1707 ± 933 | 1351 ± 1002 | 1419 ± 935 | 1160 ± 1016 | 747 ± 642 | 2394 ± 1509 |
|  | WCG | 2118 ± 1055 | 2687 ± 1357 | 1499 ± 1110 | 2799 ± 904 | 2732 ± 2788 | 1227 ± 610 | 2599 ± 2096 |
| mTT | CG | 77.2 ± 49.2 | 67.7 ± 37.7 | 76.8 ± 58 | 75.4 ± 56.5 | 123.3 ± 178.6 | 111.3 ± 114.8 | 74.2 ± 57.7 |
|  | WCG | 98.5 ± 81.8 | 91.5 ± 90.0 | 52.7 ± 21.6 | 87.5 ± 57.6 | 103.7 ± 188.7 | 111.6 ± 109.7 | 53.8 ± 31.7 |
| RT | CG | 20.0 ± 5.1 | 18.1 ± 5.2 | 17.9 ± 9.6 | 17.1 ± 4.2 | 18.5 ± 14.1 | 18.2 ± 5.6 | 22.1 ± 9.2 |
|  | WCG | 19.3 ± 5.8 | 21.8 ± 2.9 | 14.5 ± 7.0 | 19.7 ± 4.8 | 15.1 ± 4.0 | 21.6 ± 6.1 | 16.3 ± 2.7 |
| TTP | CG | 33.5 ± 7.9 | 32.7 ± 9.3^†^ | 34.4 ± 9.4 | 33.0 ± 4.4 | 33.6 ± 10.7 | 31.8 ± 5.0 | 36.7 ± 8.6 |
|  | WCG | 34.0 ± 6.4 | 34.5 ± 5.7 | 28.4 ± 6.2 | 31.6 ± 6.2 | 31.7 ± 3.6 | 36.0 ± 3.9 | 32.9 ± 4.9 |
| FT | CG | 38.1 ± 9.6 | 32.5 ± 11.0 | 32.1 ± 22.8 | 29.8 ± 10.6 | 40.3 ± 45.7 | 34.4 ± 15.8 | 44.6 ± 26.1 |
|  | WCG | 36.0 ± 14.8 | 43.7 ± 9.3 | 27.3 ± 21.3 | 40.4 ± 15.0 | 26.0 ± 12.8 | 43.3 ± 20.0 | 27.0 ± 7.0 |
| WiR | CG | 5.2 ± 3.8 | 10.1 ± 10.5 | 9.9 ± 12.0 | 6.5 ± 2.9 | 14.5 ± 26.3 | 2.8 ± 1.6 | 7.8 ± 7.7 |
|  | WCG | 8.6 ± 7.2 | 6.9 ± 4.2 | 11.1 ± 7.4 | 9.4 ± 4.2 | 18.0 ± 14.0 | 4.3 ± 3.0 | 13.1 ± 9.1 |
| WiPI | CG | 46.6 ± 36.4 | 65.4 ± 51.0 | 56.2 ± 57.8 | 47.5 ± 20.5 | 43.2 ± 27.1 | 20.9 ± 10.9 | 58.8 ± 33.9 |
|  | WCG | 66.8 ± 43.9 | 63.9 ± 32.0 | 63.7 ± 45.7 | 75.2 ± 18.9 | 116.7 ± 97.2 | 35.0 ± 17.4 | 96.8 ± 63.2 |
| WoR | CG | 2.2 ± 1.8 | 5.2 ± 6.1 | 5.9 ± 7.2 | 3.4 ± 1.9 | 11.4 ± 23.7 | 1.3 ± 0.9 | 4.1 ± 5.1 |
|  | WCG | 4.4 ± 4.5 | 2.7 ± 1.5 | 6.3 ± 4.4 | 4.2 ± 2.8 | 10.2 ± 7.8 | 2.2 ± 2.1 | 7.0 ± 5.5 |
| Data are presented as mean ± SD of 8 beagles per group for all time points. Beagles in the control group (n = 8) were fed to maintain an ideal body weight throughout the study. The weight-change group (n = 8) was fed to develop obesity (week 0 – 47), to maintain a stable body weight (week 47 – 56) and to lose weight (week 56 -83). ^a^Data from one dog missing at week 24 and week 83. PE, peak enhancement; WiAUC, wash-in area under the curve; AUC, total area under the curve; WoAUC, wash-out area under the curve; mTT, mean transit time; RT, rise time; TTP, time-to-peak; FT, fall time; WiR, wash-in rate; WiPI, wash-in perfusion index; WoR, wash-out rate. ^*^P = 0 - 0.001, ^§^P = 0.001 - 0.01 and ^†^P = 0.01 - 0.05, after multiple correction, for the group x time interaction. | | | | | | | | |
